# Role of Constitutive STAR in Leydig Cells

**DOI:** 10.3390/ijms22042021

**Published:** 2021-02-18

**Authors:** Melanie Galano, Yuchang Li, Lu Li, Chantal Sottas, Vassilios Papadopoulos

**Affiliations:** Department of Pharmacology and Pharmaceutical Sciences, School of Pharmacy, University of Southern California, Los Angeles, CA 90089, USA; galano@usc.edu (M.G.); yuchangl@usc.edu (Y.L.); lli172@usc.edu (L.L.); sottas@usc.edu (C.S.)

**Keywords:** STAR, cholesterol, lipid droplets, steroidogenesis, diacylglycerol

## Abstract

Leydig cells contain significant amounts of constitutively produced steroidogenic acute regulatory protein (STAR; STARD1). Hormone-induced STAR plays an essential role in inducing the transfer of cholesterol into the mitochondria for hormone-dependent steroidogenesis. STAR acts at the outer mitochondrial membrane, where it interacts with a protein complex, which includes the translocator protein (TSPO). Mutations in STAR cause lipoid congenital adrenal hyperplasia (lipoid CAH), a disorder characterized by severe defects in adrenal and gonadal steroid production; in Leydig cells, the defects are seen mainly after the onset of hormone-dependent androgen formation. The function of constitutive STAR in Leydig cells is unknown. We generated STAR knockout (KO) MA-10 mouse tumor Leydig cells and showed that STAR KO cells failed to form progesterone in response to dibutyryl-cAMP and to TSPO drug ligands, but not to 22(*R*)-hydroxycholesterol, which is a membrane-permeable intermediate of the CYP11A1 reaction. Electron microscopy of STAR KO cells revealed that the number and size of lipid droplets were similar to those in wild-type (WT) MA-10 cells. However, the density of lipid droplets in STAR KO cells was drastically different than that seen in WT cells. We isolated the lipid droplets and analyzed their content by liquid chromatography–mass spectrometry. There was a significant increase in cholesteryl ester and phosphatidylcholine content in STAR KO cell lipid droplets, but the most abundant increase was in the amount of diacylglycerol (DAG); DAG 38:1 was the predominantly affected species. Lastly, we identified genes involved in DAG signaling and lipid metabolism which were differentially expressed between WT MA-10 and STAR KO cells. These results suggest that constitutive STAR in Leydig cells is involved in DAG accumulation in lipid droplets, in addition to cholesterol transport. The former event may affect cell functions mediated by DAG signaling.

## 1. Introduction

Steroidogenesis begins with the transport of cholesterol from intracellular stores into the mitochondria. This is the hormone-sensitive and rate-limiting step. The steroidogenic acute regulatory protein (STAR; STARD1) plays a critical role in cholesterol transport to the mitochondria for steroidogenesis. In Leydig cells, STAR is constitutively expressed under basal conditions, independent of hormonal stimulation, which parallels the formation of low steroid levels [1]. Hormonal stimulation causes a rapid induction of STAR, which is coupled to an increase in cholesterol transfer to mitochondria and increased steroid formation [2]. It has been suggested that STAR along with other cytosolic and outer mitochondrial membrane proteins such as the translocator protein (TSPO; 18 kDa) form the transduceosome complex upon hormonal stimulation to move cholesterol to the inner mitochondrial membrane, where the CYP11A1 enzyme resides and converts cholesterol into pregnenolone [3].

STAR belongs to a family of structurally related proteins that contains the STAR-related lipid transfer (START) domain, of which STAR is the only member shown to be involved in cholesterol transfer for steroidogenesis [4]. STAR is synthesized as a 37 kDa active cytosolic protein, and upon hormonal stimulation, it moves to the outer mitochondrial membrane, where it functions in steroidogenesis and where its mitochondrial targeting sequence is cleaved, yielding an inactive 30 kDa protein [5]. Although the exact mechanism by which STAR induces steroidogenesis remains heavily debated, its critical role in cholesterol transport and steroid formation is widely accepted due to observations from STAR knockout (KO) mouse models, which accumulate lipids in adult adrenals and gonads and require adrenal steroid replacement for survival [6]. These findings support the phenotype seen in patients with lipoid congenital adrenal hyperplasia (lipoid CAH), a disease caused by mutations in the human *Star* gene and characterized by severe deficiency in steroid production and lipid accumulation in steroidogenic cells [7].

Despite current evidence revealing the importance of STAR’s function in cholesterol transfer and steroidogenesis, no studies have been conducted specifically examining the role of constitutive STAR. However, previous studies have suggested a dual functionality for STAR, with one role independent of cholesterol transport [8,9]. To study the potential roles of constitutive STAR, we developed STAR KO MA-10 mouse tumor Leydig cells. STAR KO cells do not produce steroids in response to hormonal stimulation but do respond to treatment with 22(*R*)-hydroxycholesterol. Moreover, TSPO drug ligands, which induce steroidogenesis independent of hormonal stimulation, do not induce progesterone production in STAR KO cells [10,11]. Our structural and functional studies revealed alterations in lipid droplet content in STAR KO cells. The increase in cholesterol esters (CE) found in STAR KO lipid droplets and the accumulation of diacylglycerol (DAG) and phosphatidylcholine (PC) suggest that basal STAR may function independent of cholesterol transport, including lipid droplet biogenesis. Collectively, our results show that basal STAR has a function distinct from hormone-induced STAR, which involves DAG metabolism or signaling in lipid droplets.

## 2. Results

### 2.1. Induction of Constitutive Star following Hormonal Stimulation

**Figure 1:**
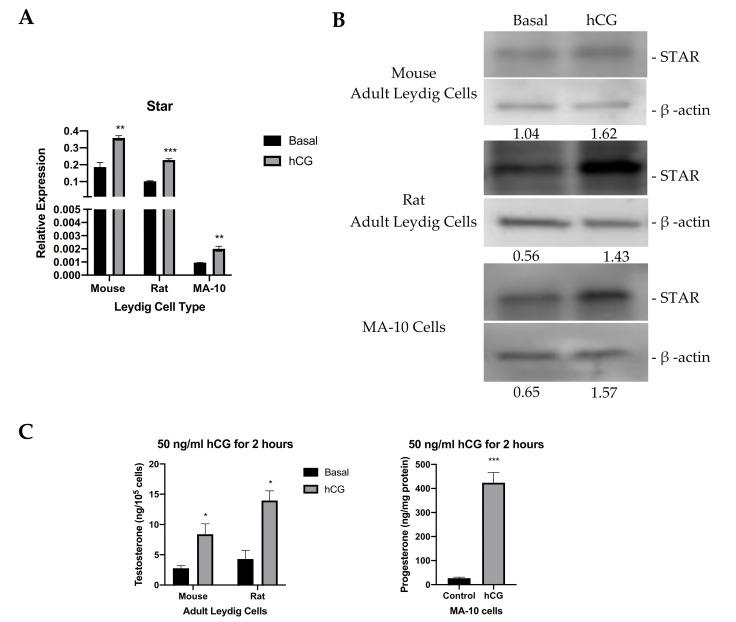
STAR expression and levels in various models in the absence and presence of hormonal stimulation. (**A**). qRT-PCR analyses of *Star* mRNA expression in mouse, rat, and MA-10 Leydig cells under basal conditions and upon hCG stimulation. *Gapdh* was used as the housekeeping gene. Data are shown as the mean ± SEM (*n* = 3). (**B**). Western blot analyses of STAR protein levels in mouse, rat, and MA-10 Leydig cells under basal conditions and upon hCG stimulation. Quantification was performed by calculating the ratio of the density of the STAR band to that of β-actin through ImageJ. Quantification is shown below each immunoblot. (**C**). ELISA analyses of testosterone production by mouse and rat Leydig cells (left) and progesterone production by MA-10 cells (right) in cell media following stimulation with 50 ng/mL hCG for 2 h. Data are shown as the mean ± SEM (*n* = 3 for mouse; *n* = 2 for rat). * *p* < 0.05; ** *p* < 0.01; *** *p* < 0.001.

Steroidogenic activity of MA-10 cells, and their responsiveness to hormones vary over time. Therefore, it is essential to our work to assess steroidogenic capacity and the responsiveness of our cells to hormonal stimulation, and in this case, STAR expression levels. To do so, we compared STAR expression levels and steroidogenic activity in MA-10 cells to those of adult mouse and rat Leydig cells. Leydig cells were isolated from adult mouse and rat testes. Figure 1A shows *Star* mRNA expression in mouse and rat Leydig cells and in WT MA-10 cells under basal conditions and upon hormonal stimulation. In all models, *Star* mRNA is present under basal conditions, and stimulation with 50 ng/mL hCG causes an induction of *Star* mRNA. STAR protein levels were also analyzed from mouse and rat Leydig cells and in WT MA-10 cells. In correlation with the quantitative real-time PCR (qRT-PCR) data, the immunoblots in Figure 1B show the protein levels of basal STAR in all models, which are all increased after stimulation with hCG. Testosterone production by isolated adult mouse and rat Leydig cells and progesterone production by MA-10 cells under basal conditions and in response to 50 ng/mL hCG were also measured, showing an increase in steroid production following hormonal stimulation (Figure 1C). In addition to characterizing the responsiveness of our MA-10 cells, these data show that, in Leydig cells, hormonal stimulation parallels an increase in STAR expression and in steroid production, but that STAR expression and low levels of steroid production are also present under basal conditions. This suggests a possible role of constitutively expressed STAR independent of hormonal stimulation.

### 2.2. Gene Deletion of Star by CRISPR/Cas9

**Figure 2:**
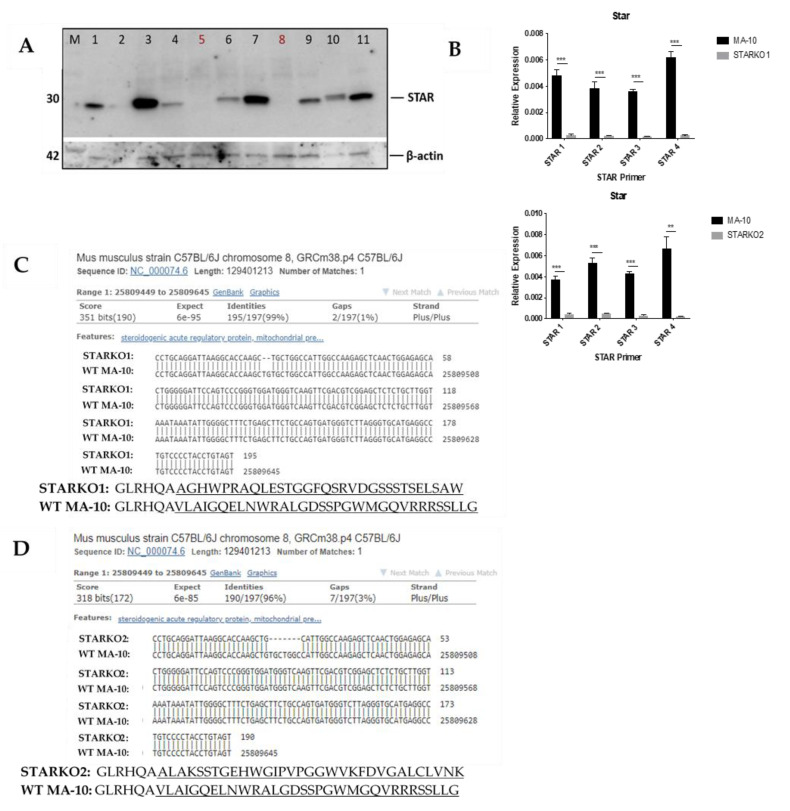
Screening and validation of CRISPR/Cas9-mediated STAR KO in MA-10 cells. (**A**) Western blot screening for STAR KO cell line following CRISPR/Cas9-mediated STAR KO and FACS. Samples in lane 5 (STARKO2) and lane 8 (STARKO1) show no STAR band and, therefore, are STAR KOs. (**B**) qRT-PCR analyses of STARKO1 and STARKO2 where *Gapdh* was used as the housekeeping gene. STAR 1, STAR 2, STAR 3, and STAR 4 are primers for various regions of the mouse *Star* gene. Data are shown as the mean ± SD (*n* = 3). ** *p* < 0.01; *** *p* < 0.001. (**C**) BLAST DNA sequence of *Star* in STARKO1 cells (top) compared to wild-type mouse Star (bottom). Dashes represent nucleotide deletions. Amino acid sequence of wild-type STAR (top) compared to that of STARKO1 (bottom). Highlighted amino acids represent sequence changes. (**D**) BLAST DNA sequence of *Star* in STARKO2 cells (top) compared to wild-type mouse Star (bottom). Dashes represent nucleotide deletions. Amino acid sequence of wild-type STAR (top) compared to that of STARKO2 (bottom). Underlined amino acids represent sequence changes.

To develop stable STAR KO cell lines from MA-10 cells, guide RNAs (gRNAs) were designed to specifically target part of exon2 of *Star*, which corresponds to STAR’s N-terminal mitochondrial targeting sequence. Two gRNAs were generated using the following single-stranded (ss) oligonucleotides: (1) F: 5′-ATTAAGGCACCAAGCTGTGCGTTTT-3′ and R: 5′-GCACAGCTTGGTGCCTTAATCGGTG-3′ (2) F: 5′-CACCAAGCTGTGCTGGCCATGTTTT-3′ and R: 5′-ATGGCCAGCACAGCTTGGTGCGGTG-3′. The gRNAs were each introduced into plasmids containing the Cas9 endonuclease and orange fluorescent protein (OFP). These plasmids were then transfected into MA-10 cells. OFP-positive cells were fluorescence-activated cell (FAC) sorted into single cells per well and cultured until enough protein could be extracted from the cells to screen for STAR KO by immunoblot. Various protein samples from single colonies that were probed for the presence of STAR are shown in Figure 2A. This analysis showed that the STAR protein was absent in samples 5 and 8, which were subsequently named STARKO2 cells and STARKO1 cells, respectively.

Lack of gene expression of *Star* in STARKO1 and STARKO2 KO cell lines was further validated by qRT-PCR, the results of which are shown in Figure 2B. *Star* mRNA expression was significantly decreased in both STARKO1 and STARKO2 for all primers used (STAR 1–4), which were specific for different regions of the *Star* gene. Next-generation sequencing (NGS)-based amplicon sequencing and Basic Local Alignment Search Tool (BLAST) results revealed that CRISPR/Cas9 introduced a frameshift mutation in both STAR KO cell lines that disrupted the *Star* gene (Figure 2C,D). We found that STARKO1 had a deletion of two nucleotides in exon2, resulting in a frameshift mutation and complete disruption of the *Star* gene sequence following the deletions. This corresponded with a change in the amino acid sequence of STAR following these mutations (Figure 2C). Similarly, using the NGS results and BLAST to align STARKO2 with WT *Star*, we found that there were seven nucleotides deleted from the *Star* gene in STARKO2, again resulting in frameshift mutations and changes in the amino acid sequence of STAR (Figure 2D). These results suggest that STARKO1 and STARKO2 are true STAR KO cell lines, where the *Star* gene is mutated beginning at exon2.

### 2.3. STAR KO Inhibits Hormone-Induced Steroidogenesis

**Figure 3:**
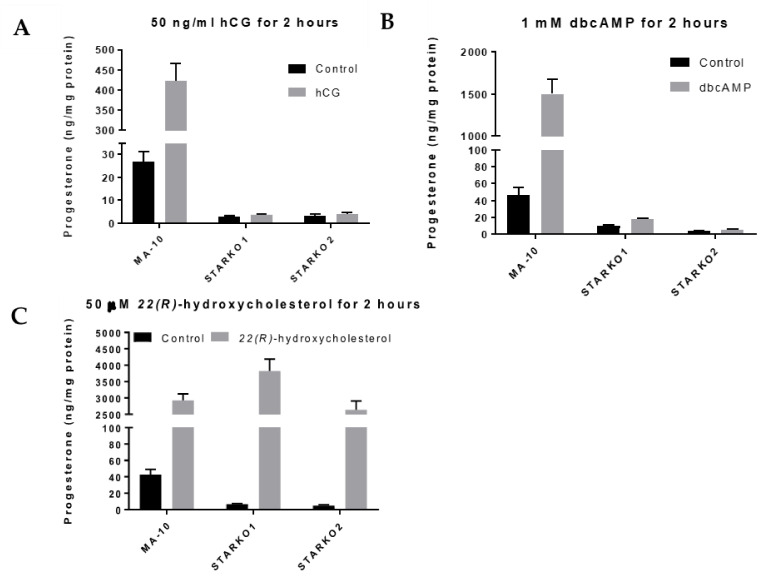
Progesterone production by wild-type MA-10 and STAR KO cell lines in response to stimulus. (**A**) ELISA analyses of progesterone levels in cell media following stimulation with 50 ng/mL hCG for 2 h. (**B**) ELISA analyses of progesterone levels in cell media following stimulation with 1 mM dbcAMP for 2 h. (**C**) ELISA analyses of progesterone levels in cell media following stimulation with 50 μM 22(*R*)-hydroxycholesterol for 2 h. Data are shown as the mean ± SEM (*n* = 3).

To determine whether STAR activity was disrupted, we used ELISA to measure progesterone levels produced by STARKO1 and STARKO2 cells in response to hormonal stimulation. Neither STARKO1 nor STARKO2 could be induced to produce progesterone in response to hCG stimulation (Figure 3A). This suggests that the function of STAR in cholesterol transport, which is critical in hormone-induced steroidogenesis, is disrupted as a result of our STAR KO. Similar trends were seen for both STAR KO cell lines following stimulation with dbcAMP (Figure 3B).

To ensure that the steroidogenic pathway downstream of STAR was not affected as a result of STAR KO, we treated the STAR KO cells with 22(*R*)-hydroxycholesterol, a membrane-permeable intermediate of the CYP11A1 (P450scc) reaction, which is downstream of STAR activity. Treatment with 22(*R*)-hydroxycholesterol in STARKO1 and STARKO2 produced progesterone at levels similar to WT MA-10 cells (Figure 3C). These results suggest that the steroidogenic pathway downstream of STAR activity was still intact in both STAR KO cell lines. Because STARKO1 and STARKO2 act identically in response to these various treatments, we chose to carry out subsequent experiments using only STARKO1.

### 2.4. STAR KO Inhibits TSPO Ligand-Mediated Steroidogenesis

**Figure 4:**
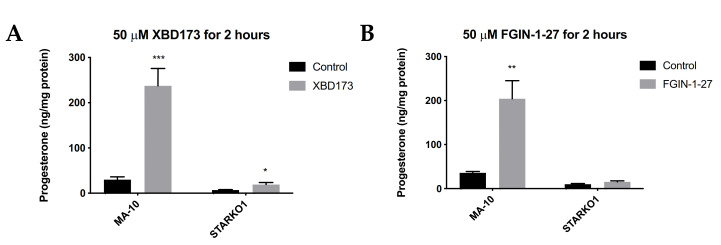
Progesterone production by wild-type MA-10 and STAR KO cell lines in response to TSPO drug ligands. (**A**). ELISA analyses of progesterone levels in cell media following stimulation with 50 μM XBD173 for 2 h. (**B**). ELISA analyses of progesterone levels in cell media following stimulation with 50 μM FGIN-1-27 for 2 h. Data are shown as the mean ± SEM (*n* = 3). * *p* < 0.05; ** *p* < 0.01; *** *p* < 0.001.

We next sought to determine whether STAR KO affects TSPO-mediated steroidogenesis. TSPO drug ligands such as XBD173 and FGIN-1-27 have been shown to induce steroidogenesis in the absence of hormonal stimulation, but in the presence of basal levels of STAR [10,11]. Since STAR KO cells lack constitutive STAR, we sought to determine whether TSPO ligand-induced steroidogenesis acts independently of STAR. MA-10 and STAR KO cells were treated with 50 μM XBD173 or 50 μM FGIN-1-27 for 2 h and progesterone production was measured by ELISA. Results showed that XBD173 stimulates progesterone production in MA-10 cells, but that progesterone production is not induced to levels similar to WT MA-10 cells when STAR is absent (Figure 4A). Although progesterone production was not induced in STARKO1 cells to the extent of WT MA-10 cells, XBD173 caused a small but significant increase in progesterone produced by STARKO1 cells compared to control STARKO1 cells (Figure 4A). Treatment with FGIN-1-27 also showed that progesterone production is not induced to levels similar to WT MA-10 cells when STAR is absent (Figure 4B). However, there was no difference in progesterone produced between control and FGIN-1-27-treated STARKO1 cells. These results suggest that constitutive STAR, or STAR that is present independent of hormonal stimulation in MA-10 cells but absent in STAR KO cells, plays a role in TSPO-mediated steroidogenesis.

### 2.5. STAR KO Results in Alterations in Lipid Droplet Content

**Figure 5:**
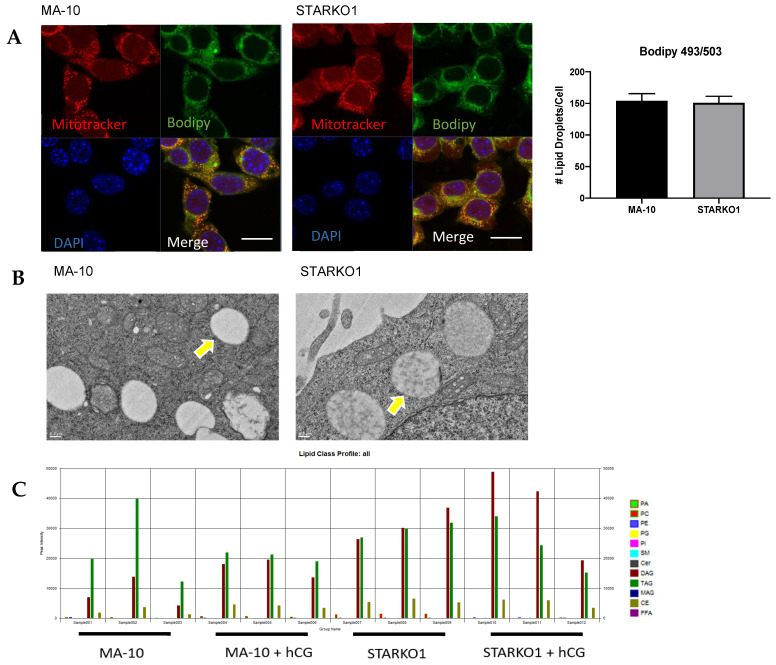

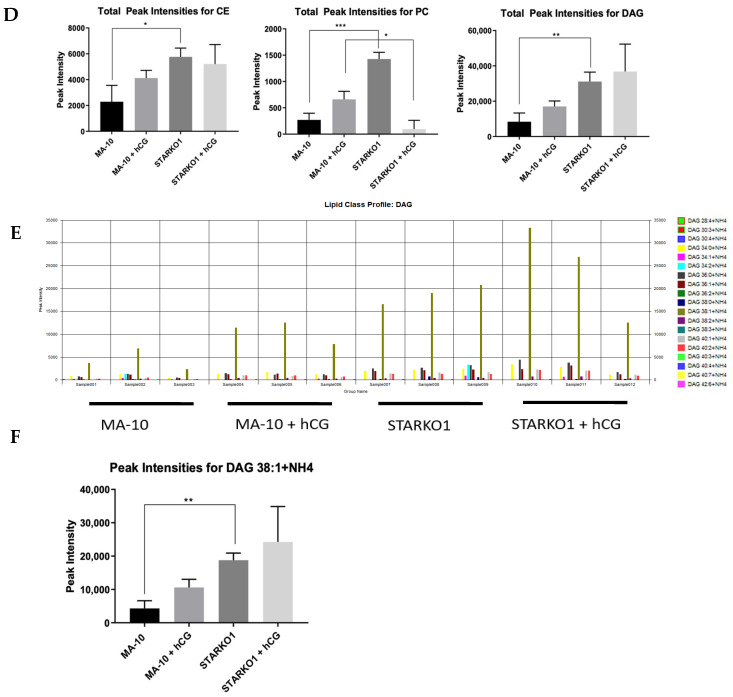
Alterations in lipid droplet content between WT MA-10 and STARKO1 cells. (**A**) Bodipy 493/503 staining of lipid droplets in MA-10 (left) and STARKO1 (right) cells with quantification shown to the right. Scale bar: 10 μm. (**B**) Electron microscopy images of lipid droplets in WT MA-10 cells (left) and STARKO1 cells (right). Arrows point to lipid droplets. (**C**) Peak intensities of various lipid classes identified in lipid droplets of wild-type MA-10 and STARKO1 cells in the absence and presence of hormonal stimulation. (**D**) Peak intensities of lipid classes shown to be significantly altered between lipid droplets of wild-type MA-10 and STARKO1 cells in the absence and presence of hormonal stimulation. (**E**) Peak intensities of individual diacylglycerol (DAG) species identified in lipid droplets of MA-10 and STARKO1 cells. (**F**) Peak intensities of DAG 38:1 in lipid droplets of MA-10 and STARKO1 cells in the absence and presence of hormonal stimulation. Data are shown as the mean ± SEM. * *p* < 0.05; ** *p* < 0.01; *** *p* < 0.001.

A distinctive characteristic of Leydig cells is the abundance of large lipid droplets. Bodipy 493/503 staining showed no difference in lipid droplet number per cell between WT MA-10 and STARKO1 cells (Figure 5A). Electron microscopy of STAR KO cells also revealed that the number and size of lipid droplets were similar to those in WT MA-10 cells (Figure 5B). The lack of lipid droplet accumulation in STARKO1 cells is in contradiction to the proposed role of STAR in lipoid CAH. Our data suggest that basal STAR expression may have a function different than hormone-induced STAR since unstimulated STAR KO cells do not accumulate lipid droplets. While there was no difference in lipid droplet number, the density of the lipid droplets in STAR KO cells was drastically different than that seen in WT cells (Figure 5B). Lower magnification electron micrographs are shown in Figure S1 to provide a better overview of changes in density between the lipid droplets of WT MA-10 and STAR KO cells.

Liquid chromatography–mass spectrometry (LC-MS) data showed differences in the lipid profiles of MA-10 and STARKO1 cell lipid droplets (Figure 5C). There was a significant increase in CE and PC in STAR KO cell lipid droplets, but the most abundant increase was in the amount of DAG (Figure 5D). There were no significant differences in DAG and CE content between WT and STAR KO cells upon hCG stimulation, but there was a significant change in PC content (Figure 5D). The profile of individual DAG species revealed that DAG 38:1 is the species that is primarily increased in STARKO1 lipid droplets compared to WT (Figure 5E,F). These results suggest that DAG accumulation in lipid droplets and signaling may play a role in the pathology of lipoid CAH, including loss of the ability to form steroids as seen with *STAR* mutations in humans. The differences in lipid droplet content between WT and STAR KO MA-10 cells in the absence and presence of hormonal stimulation also suggest that STAR may have a function in lipid metabolism that is independent of its role in cholesterol transport.

### 2.6. DAG Signaling May Play a Role in Progesterone Production

**Figure 6:**
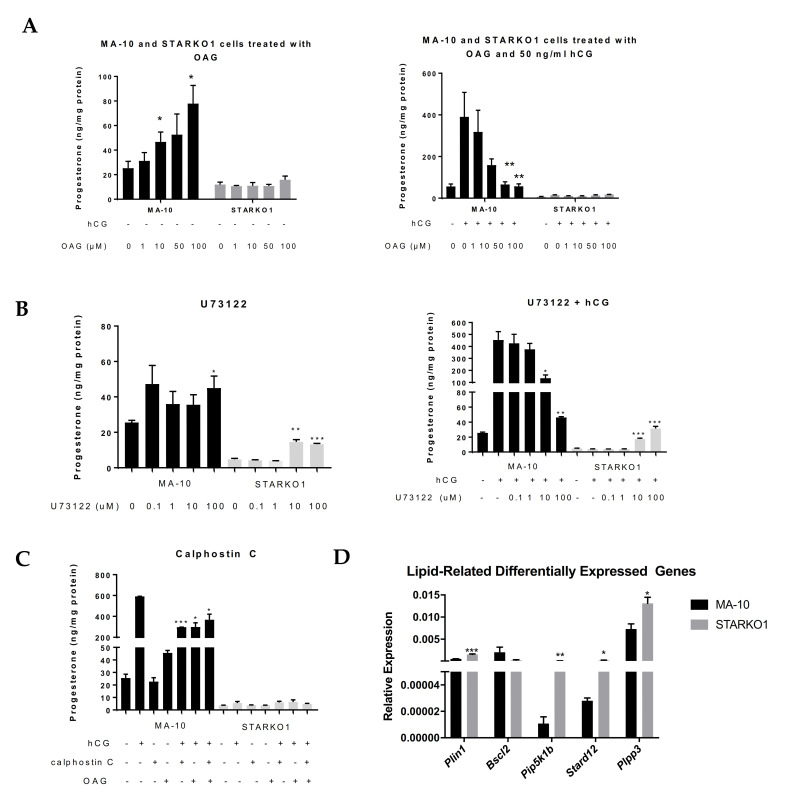
Role of DAG signaling in progesterone production by WT MA-10 and STARKO1 cells. (**A**) ELISA analyses of progesterone levels in cell media following treatment with varying concentrations of the DAG analog OAG in the absence (left) and presence (right) of 50 ng/mL hCG for 2 h. (**B**) ELISA analyses of progesterone levels in cell media following treatment with varying concentrations of PLC inhibitor U73122 in the absence (left) and presence (right) of 50 ng/mL hCG for 2 h. (**C**) ELISA analyses of progesterone levels in cell media following treatment with 1 μM of PKC inhibitor calphostin C in combination with 50 ng/mL hCG and/or 50 μM OAG for 2 h. (**D**) qRT-PCR analyses of lipid-related differentially expressed genes. Data are shown as the mean ± SEM (*n* = 3). * *p* < 0.05; ** *p* < 0.01; *** *p* < 0.001.

To explore the effects of DAG accumulation when STAR is knocked out, we treated MA-10 and STARKO1 cells with the DAG analog, 1-oleoyl-2-acetyl-*sn*-glycerol (OAG) for two hours in the absence and presence of hormonal stimulation. Treatment with OAG led to a dose-dependent increase in basal progesterone production but inhibition of hormone-induced progesterone production in MA-10 cells (Figure 6A). However, OAG had no effect on either the basal or hormone-stimulated progesterone formation by STARKO1 cells (Figure 6A). This further suggests that the increase in DAG accumulation seen in the lipid droplets of STAR KO cells may play a role in the inability of STAR KO cells to produce steroids. To further investigate the effects of DAG alterations, we inhibited phospholipase C (PLC) and protein kinase C (PKC), which are upstream and downstream of DAG, respectively, in the DAG signaling pathway. Surprisingly, treatment with the PLC inhibitor U73122 in the absence and presence of 50 ng/mL hCG for two hours significantly increased progesterone production at 10 and 100 μM in STARKO1 cells (Figure 6B). In WT MA-10 cells, treatment with these concentrations of U73122 in combination with 50 ng/mL hCG significantly decreased progesterone production (Figure 6B). However, treatment with the PKC inhibitor calphostin C (1 μM) alone or in combination with 50 ng/mL hCG and/or 50 μM OAG for two hours, did not alter progesterone production in STARKO1 cells (Figure 6C), suggesting that DAG itself, rather than its effect on PKC, may inhibit steroid production in STARKO1 cells. Collectively, these data suggest that DAG may play a role in the inhibition of steroidogenesis when STAR is knocked out, which may be attributed to the activation of PLC but is independent of DAG activation of PKC.

To gain further insight into the function of STAR in lipid metabolism or DAG signaling, we carried out RNA sequencing to identify differentially expressed genes between MA-10 and STARKO1 cells related to lipids and/or DAG (data not shown). Among the differentially expressed genes identified by Ingenuity Pathway Analysis, some genes were part of lipid and DAG interaction networks. These genes include *Plin1, Bscl2, Pip5k1b, Stard12, and Plpp3*. These genes were confirmed to be significantly differentially expressed by qRT-PCR except *Bscl2*, which was decreased in STARKO1 cells, but not significantly (Figure 6D). Perilipin 1 (PLIN1) and Berardinelli-Seip congenital lipodystrophy 2/Seipin (BSCL2) are lipid droplet-associated proteins [12,13]. STARD12 (DLC-1) is a member of the START protein family; phosphoinositide 5-kinase 1 β (PIP5K1β) is important in phospholipase D (PLD) activation; and phospholipid phosphatase 3 (PLPP3/LPP3) plays a role in DAG formation [14,15,16]. These alterations in gene expression among genes involved in lipid metabolism and DAG signaling further demonstrate that STAR in MA-10 cells seems to have a function independent of cholesterol transport for steroidogenesis.

## 3. Discussion

In Leydig cells, basal levels of STAR correlate with low levels of steroidogenesis [1]. Hormonal stimulation causes a rapid increase in *Star* mRNA expression and STAR protein levels, which parallels an induction of steroidogenesis [2]. Multiple lines of evidence have shown that STAR plays a critical function in cholesterol transport into the mitochondria for hormone-induced steroidogenesis [17,18,19,20]. Indeed, STAR overexpression in MA-10 cells induces progesterone production to similar levels as those seen in response to cAMP stimulation [17]. Furthermore, transient transfection of *Star* cDNA, in addition to that of the CYP11A1 system, into non-steroidogenic cells increased pregnenolone synthesis greater than 4-fold [18]. The importance of STAR in cholesterol transport is most evident through the presence of *STAR* mutations in humans which causes lipoid CAH [19]. Despite evidence supporting the role of STAR in cholesterol transport and hormone-induced steroidogenesis, the exact function of constitutively expressed STAR remains unknown. Previous work in our laboratory identified small-molecule inhibitors of the cholesterol-binding domain of STAR, where even the most active compound only inhibited steroidogenesis by about 60% [8]. Additionally, a study that blocked phosphorylation of an important phosphorylation site on the cholesterol-binding domain of STAR only resulted in 50% inhibition of its activity [9]. These findings suggest a cholesterol-independent function of STAR, since blocking its cholesterol-binding domain does not completely abolish steroidogenic activity.

The current study provides evidence that constitutive STAR may have a function which is distinct from that of hormone-induced STAR. It is important to note that the data presented here suggest that constitutive STAR may have a role distinct from hormone-induced STAR in MA-10 mouse tumor Leydig cells and, at present, it is unknown whether this role may be consistent among normal Leydig cells and those from other species. Further investigation should be performed to elucidate the role of constitutive STAR in steroidogenic cells of other species, such as in Leydig cells of rats, since the importance of lipid droplets as a source of cholesterol for steroidogenesis varies between species. To study the potential function(s) of constitutive STAR, we used CRISPR/Cas9 to develop a STAR KO MA-10 cell line (STARKO1), which does not respond to hormonal stimulation or treatment with TSPO drug ligands. TSPO drug ligands have previously been shown to induce steroidogenesis independent of hormonal stimulation, but in the presence of constitutive STAR [10,11]. Since TSPO drug ligands are unable to induce steroid formation in STAR KO cells, this suggests that basal STAR functions in TSPO-mediated steroidogenesis. The limited increase in progesterone produced by STARKO1 cells treated with XBD173 may be due to STAR-independent mechanisms of steroidogenesis. 

It is currently thought that mutant *STAR* in humans inhibits acute steroidogenesis and that continued hormonal stimulation leads to the accumulation of cholesterol as CE in the lipid droplets of steroidogenic cells, causing cell damage and an inhibition of basal steroidogenesis [20]. Our data suggest that STAR KO cells do not accumulate lipids, but that the contents of their lipid droplets are different than WT cells. This contradicts the proposed role of STAR in lipoid CAH, suggesting that basal STAR may have a function different than hormone-induced STAR, since the absence of constitutive STAR in STAR KO MA-10 cells does not alter lipid droplet number. Additionally, while STAR KO MA-10 cells do not accumulate lipid droplets, previous work utilizing a single-cell CRISPR/Cas9 approach to delete STAR in Y-1 adrenal cells showed that transfected (CRISPR-positive) cells accumulate lipid droplets 24 and 48 h after transfection [21]. The discrepancy between our data and this previous study may be due to tissue-specific differences, evident through work showing that lipid droplet accumulation in the adrenals of STAR KO mice is significantly more severe compared to lipid droplet accumulation seen in STAR KO mouse testes [6]. It is also possible that the generation of a stable STAR KO cell line as described here may give more insight to the long-term effects of STAR KO compared to a transient transfection.

We investigated the contents of the lipid droplets and found that the lipid profiles of the lipid droplets between WT MA-10 and STARKO1 cells were different. The most abundant change was an increase in the amount of DAG in STARKO1 cell lipid droplets, which suggests that STAR may play a role in lipid metabolism and/or DAG signaling. The levels of CE and PC in STARKO1 cell lipid droplets were also significantly higher than those seen in WT MA-10 lipid droplets. Accumulation of CE in STARKO1 lipid droplets was expected as the absence of STAR would increase free cholesterol to be converted into CE and stored in lipid droplets. The increase in PC may explain the increase in DAG levels, as PC can be converted to DAG through a PLC-dependent mechanism in addition to a PLD-dependent mechanism [22].

To explore possible implications of DAG accumulation in STARKO1 lipid droplets, we treated MA-10 and STARKO1 cells with various molecules involved in DAG signaling. The dose-dependent inhibition of hormone-induced steroid production in MA-10 cells by OAG demonstrates that DAG accumulation may play a role in the lack of steroid production when STAR is knocked out. Previous work has demonstrated that STAR overexpression in high-fat diet-induced non-alcoholic fatty liver disease (NAFLD) in mice decreased intracellular DAG levels, indicating a protective role for STAR in NAFLD through a reduction in DAG levels [23]. Since this shows that STAR overexpression may lead to a reduction in DAG levels, it is consistent with our data which demonstrate that STAR KO leads to an accumulation of DAG. Next, we found that inhibition of PLC, which is an upstream activator of DAG, by U73122 inhibited progesterone production in MA-10 cells, but significantly increased progesterone production in STARKO1 cells. This is consistent with previous findings that showed that inhibition of PLC by U73122 in R2C rat tumor Leydig cells, which constitutively produce steroids and have high basal levels of STAR, was inhibitory to steroid production and decreased STAR protein levels [24]. The effects of DAG and the PLC inhibitor on MA-10 cells are in agreement with previous studies on the role of PKC and PLC in steroidogenic cells [25,26,27]. The increase in steroid production in STARKO1 cells in response to PLC inhibition suggests that increased activation of DAG by PLC may contribute to the lack of steroid production in the absence of STAR. This also suggests a mechanism for steroidogenesis that is STAR independent but PLC dependent. We also found that inhibition of PKC, which is activated by DAG, did not affect steroid production, suggesting that the role of STAR in DAG signaling is independent of PKC activation. This may be explained by previous data that suggested only 1,2-diacyl-*sn*-glycerols (1,2-DAG) can activate PKCs, whereas other DAG stereoisomers (1,3-diacyl-*sn*-glycerols and 2,3 diacyl-*sn*-glycerols) cannot [28,29]. Further, it has been shown that hormone-sensitive lipase (HSL) preferentially hydrolyzes *sn*-1(3) ester bonds, which then would not form 1,2-DAG and therefore not activate PKC [30]. These data further implicate STAR in DAG signaling and/or metabolism, independent of its function in cholesterol transport for hormone-induced steroidogenesis.

Lastly, we performed RNA sequencing to identify differentially expressed genes between MA-10 and STARKO1 cells. Interestingly, our RNA sequencing analysis did not show that steroidogenic factor 1 (*Sf-1*) was differentially expressed, although a previous report showed that knockout of SF-1 in Leydig cells led to lipid accumulation in part through the suppression of STAR levels [31]. However, we did identify other differentially expressed genes that are associated with lipid metabolism. PLIN1 is localized to the surface of lipid droplets and has been shown to play an essential role in regulating the accumulation and hydrolysis of triacylglyceride (TAG) and DAG in lipid droplets [13]. Upon phosphorylation by protein kinase A (PKA), PLIN1 recruits lipolytic proteins, such as HSL, which has hydrolytic activity against DAG and TAG, to the lipid droplet surface [32]. However, previous studies have demonstrated that a reduction in PLIN1 levels increased basal lipolysis but decreased PKA-stimulated lipolysis, showing that PLIN1 may function in both repressing basal lipolysis and in enhancing PKA-stimulated lipolysis [32]. Here, we see that under basal conditions, *Plin1* mRNA expression is increased when STAR is knocked out, which may contribute to decreased DAG metabolism. We also identified other genes associated with DAG formation (PLPP3 and PIP5K1β) that were found to be increased in STARKO1 cells compared to WT MA-10 cells. PLPP3 dephosphorylates phosphatidic acid (PA) to form DAG [33]. PIP5K1β is necessary for phosphatidylinositol 4,5-bisphosphate (PIP2) activity, which is not only metabolized to DAG, but is also an essential cofactor for PLD, which hydrolyzes PC to PA, and which then can be converted to DAG [15]. Increased expression of *Plpp3* and *Pip5k1b* therefore gives insight into possible mechanisms of DAG accumulation that we see in STARKO1 lipid droplets. Lastly, we found that expression of *Stard12* was also increased in STARKO1 cells. STARD12 was considered initially to function mainly as a tumor suppressor [14]. However, further studies have shown that STARD12 binds PLC-δ1 and activates hydrolysis of PIP2, which functions in DAG formation [34]. In addition, it has been shown that STARD12 is localized to the mitochondria in Huh-7 cells [35]. It is possible that in the absence of STARD1, *Stard12* expression increases to facilitate a compensatory mechanism. Collectively, these data are consistent with DAG accumulation seen in STARKO1 lipid droplets and further suggest a role of constitutive STAR in MA-10 cells.

## 4. Materials and Methods

### 4.1. Primary Leydig Cell Isolation

Leydig cells were isolated from adult Sprague–Dawley rats and C57BL/6 mice (Charles River Laboratories, Wilmington, MA, USA) as previously described [25]. The rats and mice were bred and maintained in accordance with protocols approved by the Institutional Animal Care and Use Committee of the University of Southern California (Protocol # 20791; approved on 8/30/2018). Testes were decapsulated, dissociated in 0.25 mg/mL collagenase, and shaken at 80 cycles/min at 34 °C for 15 min. Once dissociated, the seminiferous tubules were removed, and supernatant-containing cells were centrifuged at 800× *g* for 20 min. Pellets were applied to a Percoll density gradient and centrifuged at 14,000 rpm for 45 min at 4 °C. The Leydig cell-containing fraction was layered onto a BSA density gradient and centrifuged at 50× *g* for 10 min, which yielded 85% pure Leydig cells as revealed by 3β-hydroxysteroid dehydrogenase staining. For hCG stimulation, Leydig cells were treated with 50 ng/mL hCG for 2 h. Steroid production was measured using the Testosterone ELISA Kit (Cayman Chemical, Ann Arbor, MI, USA). Steroid production data were normalized to cell number. qRT-PCR and immunoblot analyses were carried out as described here.

### 4.2. Cell Culture

MA-10 cells were kindly provided by Dr. Mario Ascoli (University of Iowa, Iowa City, IA, USA). MA-10 cells were found to be negative for mycoplasma by the Mycoplasma PCR Detection Kit (Applied Biological Materials Inc., Ferndale WA, USA). WT MA-10 mouse tumor Leydig cells [36] and STAR KO cells were maintained in Dulbecco’s modified Eagle medium/F-12medium + Glutamax supplemented with 5% heat-inactivated fetal bovine serum, 2.5% heat-inactivated horse serum, and 1% penicillin/streptomycin at 37 °C and 3.5% CO_2_.

### 4.3. CRISPR/Cas9-Mediated Gene Deletion of Star in MA-10 Cells

Two sets of ss DNA oligonucleotides specifically targeting exon2 of Star were designed after using Synthego’s CRISPR single gRNA Design Tool (https://www.synthego.com). The ss oligonucleotides were annealed and cloned into the GeneArt^®^ CRISPR Nuclease Vector which contains an OFP reporter (Thermo Fisher Scientific, Waltham, MA, USA). Plasmid extraction was performed using the Zyppy Plasmid Miniprep Kit (Zymo Research, Irvine, CA, USA). Sequencing of the cloned plasmids were performed to confirm the insertion of the gRNAs. Plasmids were transfected into MA-10 cells using Lipofectamine 3000 and Opti-MEM according to the manufacturer’s recommendations. FACS was used to sort the OFP-positive cells into single colonies 24 h post-transfection.

STAR KO was determined using NGS, qRT-PCR, and immunoblotting. NGS was performed through GENEWIZ Next-Generation Sequencing Services. For *Star* mRNA determination, total RNA was extracted using the Quick-RNA MiniPrep Plus kit (Zymo Research, Irvine, CA, USA). An amount of 500 ng of isolated RNA was applied to amplify cDNA using the PrimeScript RT Master Mix (Takara Bio, Mountain View, CA, USA). qRT-PCR with Applied Biosystems PowerUP SYBR Green Master Mix (Thermo Scientific, Waltham, MA, USA) detection was performed using the qTOWER^3^ (Analyik Jena AG, Jena Germany). This method was used for all qRT-PCR data shown here. Primers for different regions of *Star* are as follows: STAR1 F: 5′TCCTCGCTACGTTCAAGCTG-3′ R: 5′-AGCTCCGACGTCGAACTT-3′, STAR2 F: 5′-TCGCTACGTTCAAGCTGTGTG-3′ R: 5′-GGCTCCGACGTCGAACTTGA-3′, STAR3 F: 5′-AGAGGTGGCTATGCAGAAGG-3′ R: 5′-CATGCGGTCCACAAGTTCTT-3′, and STAR4 F: 5′-GGAGCAGAGTGGTGTCATCA-3′ R: 5′-TGGCGAACTCTATCTGGGTC-3′. *Gapdh* was used as the housekeeping gene.

### 4.4. Immunoblot Analysis

Protein was extracted from mouse and rat primary Leydig cells, WT MA-10 cells, and STAR KO cells using RIPA buffer. Protein concentration was determined after centrifugation using the Pierce BCA Protein Assay Kit (Thermo Scientific, Waltham, MA, USA). Sodium dodecyl sulfate-polyacrylamide gel electrophoresis was performed using 7.5 μg of protein extract and a 4%–20% Tris–glycine gradient gel (Bio-Rad, Hercules, CA, USA) and the resulting bands electro-transferred to a polyvinylidene fluoride membrane. Blocking of the membranes was performed using 5% bovine serum albumin before incubating with STAR antibody (1:1000; Cell Signaling, Danvers, MA, USA) and secondary antibody, WesternSure^®^ Goat anti-Rabbit HRP Secondary Antibody (1:5000; LI-COR, Lincoln, NE, USA). Membranes were stripped using the Restore Western Blot Stripping Buffer (Thermo Scientific, Waltham, MA, USA) and reprobed using anti-β-actin (1:5000). The immunoreactive proteins were visualized using Radiance Peroxide and Radiance Plus (Azure Biosystems, Dublin, CA, USA) and imaged using the Azure c600 (Azure Biosystems).

### 4.5. Quantification of Steroid Production

WT MA-10 and STAR KO cells (1×10^4^ per well) were plated on 96-well plates in triplicate for 24 h. Before stimulation, medium was removed, each well was washed with phosphate-buffered saline, and serum-free medium was added with one of the following: 50 ng/mL human chorionic gonadotropin (hCG; National Hormone and Peptide Program, Harbor-UCLA Medical Center, Torrance, CA, USA), 1 mM dibutyryl cyclic AMP (dbcAMP; Sigma), 50 μM 22(*R*)-hydroxycholesterol (Sigma, St. Louis, MO, USA), 50 μM XBD173 (Sigma), 50 μM FGIN-1-27 (Cayman Chemical, Ann Arbor, MI, USA), 1-oleoyl-2-acetyl-*sn*-glycerol (Cayman Chemical, Ann Arbor, MI, USA), U73122 (Cayman Chemical, Ann Arbor, MI, USA), or 1 mM calphostin C (Cayman Chemical, Ann Arbor, MI, USA). After 2 h incubation at 37 °C, the medium was collected to measure steroid production. The remaining cells were lysed with 0.1 N sodium hydroxide for protein measurements. Steroid production was measured using the Progesterone ELISA Kit (Cayman Chemical, Ann Arbor, MI, USA). Steroid production data were normalized to protein contents.

### 4.6. Lipid Droplet Imaging, Isolation, and Analysis

Lipid droplets were imaged by transmission electron microscopy (TEM) at the Doheny Eye Institute (Los Angeles, USA). Lipid droplet isolation was performed using Cell Biolabs, Inc.’s Lipid Droplet Isolation Kit (San Diego, CA, USA). A total of 50 million cells were pelleted and processed according to the manufacturer’s recommendations. Purity of the isolated lipid droplets were assessed through immunoblots using GAPDH (1:1000; Cell Signaling) and PLIN1 (1:1000; Cell Signaling, Danvers, MA, USA) antibodies. Western blot analyses were performed as described above. The content of the isolated lipid droplets was then analyzed using LC–MS. Samples for LC–MS were prepared by adding 200 µL of water to each sample and vortexing. A volume of 1000 µL of methyl tert-butyl ether (MTBE) was then added to each sample, vortexed for 10 s and left to settle for 10 min. A volume of 800 µL of the top layers were transferred to 10 × 75 mm glass culture tubes. These steps were repeated. The MTBE extracts were then evaporated at room temperature in a Thermo Scientific™ Savant™ SPD131DDA SpeedVac™ Concentrator. Extracts were reconstituted with 250 µL 10 mM ammonium acetate in 50:50 (v:v) dichloromethane:methonol. Reconstituted extracts were then transferred to injection vials for flow infusion (no column) LC–MS analysis. LC was performed using the Shimadzu Nexera XR (Shimadzu Corporation, Kyoto, Japan) with an injection volume of 50 µL, autosampler temperature of 15 °C, and an infusion flow rate of 7.0 µL/min. MS was performed using the 5600+ TripleTOF quadrupole-time of flight system (AB SCIEX, Framingham, MA, USA). Scanning of the positive mode was performed using TOF-NS1 from m/z 200 to 1250. Scanning of the negative mode was performed through MS/MS, using unit mass resolution in the quadrupole (0.7 amu bandpass) at each nominal mass from 200–1250. MS data were analyzed using the LipidView Software (AB SCIEX, Framingham, MA, USA).

### 4.7. Mitochondria and Lipid Droplet Staining and CONFOCAL MICROSCopy

To stain mitochondria and lipid droplets, cells were plated at a density of 200,000 cells per well for 24 h and stained with 0.1 μM MitoTracker Red CMXRos (Invitrogen, Carlsbad, CA, USA) for 30 min at 37 °C, washed with PBS 3 times, and stained with 2 μM Bodipy 493/503 (Invitrogen, Carlsbad, CA, USA) for 15 min at 37 °C. Cells were fixed in 4% PFA for 15 min at room temperature, stained with DAPI, and observed by Zeiss LSM 880 confocal microscopy.

### 4.8. Statistical Analysis

All data are presented as the mean ± standard error of the mean from three independent experiments unless indicated otherwise. Moreover, all experiments were conducted in triplicate unless indicated otherwise. GraphPad Prism (version 7) was used for graphic presentation and statistical analyses of data were performed using the Student t test. Means were considered statistically different when *p* < 0.05.

## 5. Conclusions

Taken together, these results provide evidence for a function of constitutive STAR in Leydig cells that is distinct from its role in hormone-induced steroidogenesis. Constitutive STAR may function in TSPO-mediated steroidogenesis and may have a role in DAG accumulation in lipid droplets when STAR is mutated or knocked out. DAG accumulation may contribute to the inability to produce steroids in the absence of functional STAR.

## Data Availability

The data presented in this study are available in this manuscript, the Supplementary Material, and the NCBI Gene Expression Omnibus (GEO) database: accession no.: GSE165392.

## Supplementary Materials

Supplementary materials can be found at https://www.mdpi.com/1422-0067/22/4/2021/s1.

## Abbreviations

STAR	steroidogenic acute regulatory protein
TSPO	translocator protein
Lipoid CAH	lipoid congenital adrenal hyperplasia
dbcAMP	dibutyryl-cAMP
START domain	STAR-related lipid transfer domain
CE	cholesterol ester
DAG	diacylglycerol
PC	phosphatidylcholine
OFP	orange fluorescent protein
NGS	next-generation sequencing
LC–MS	liquid chromatography–mass spectrometry
OAG	1-oleoyl-2-acetyl-*sn*-glycerol
PLC	phospholipase C
PKC	protein kinase C
WT	wild type
KO	knockout
TAG	triacylglycerol
CRISPR/Cas9	clustered regularly interspaced short palindromic repeats
gRNA	guide RNA
FACS	fluorescence-activated cell sorting
BLAST	Basic Local Alignment Search Tool
PLD	phospholipase D
ss	single stranded
qRT-PCR	quantitative real-time PCR
