# Supplementary material for: Role of Constitutive STAR in Leydig Cells
Source: Int J Mol Sci. 2021 Feb 18;22(4):2021. doi: 10.3390/ijms22042021 (PMC7922663; doi:10.3390/ijms22042021)
Supplement: Supplementary file 1 [file ijms-22-02021-s001.pdf]

Role of Constitutive STAR in Leydig Cells

Melanie Galano, Yuchang Li, Lu Li, Chantal Sottas and Vassilios Papadopoulos

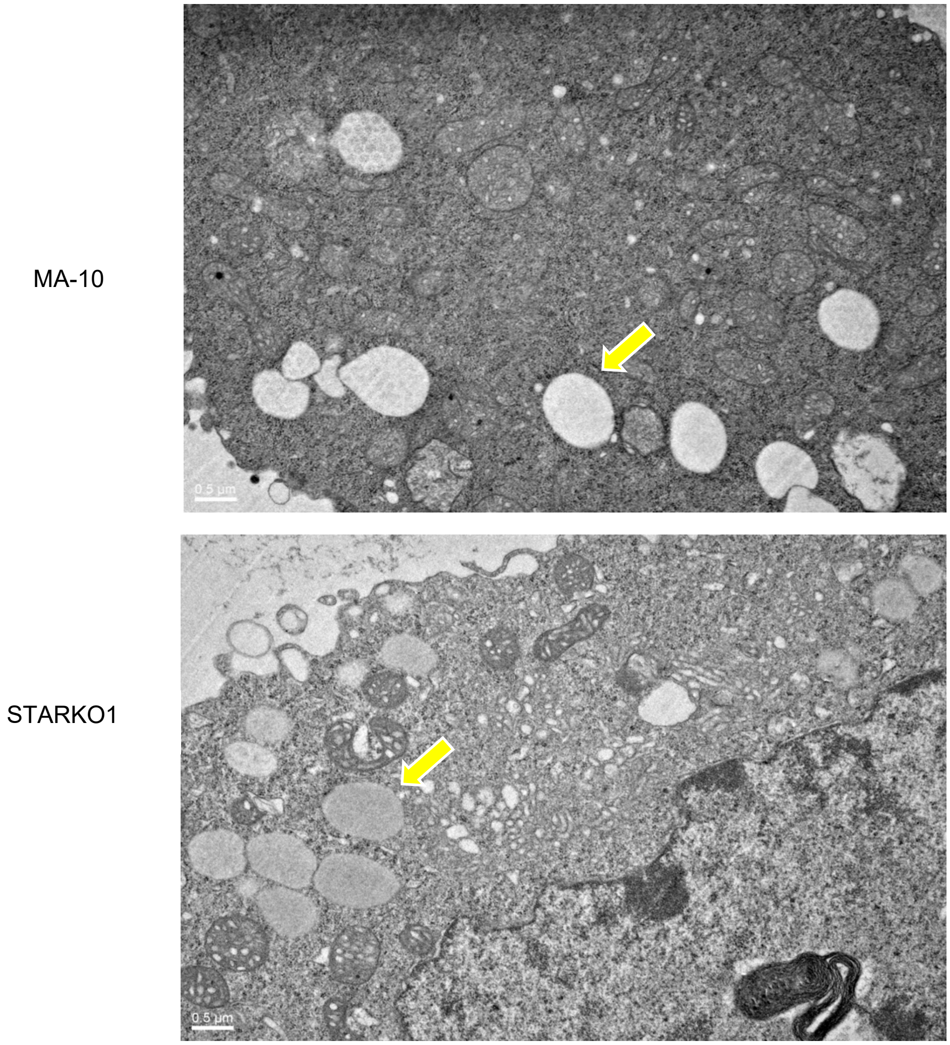

Figure S1. Electron microscopy images of lipid droplets in WT MA-10 cells (top) and STARKO1 cells (bottom). Arrows point to lipid droplets. Scale bar shows 0.5 µm.
